# Non-Additive Effects on Decomposition from Mixing Litter of the Invasive *Mikania micrantha* H.B.K. with Native Plants

**DOI:** 10.1371/journal.pone.0066289

**Published:** 2013-06-20

**Authors:** Bao-Ming Chen, Shao-Lin Peng, Carla M. D’Antonio, Dai-Jiang Li, Wen-Tao Ren

**Affiliations:** 1 State Key Laboratory of Biocontrol, School of Life Sciences, Sun Yat-Sen University, Guangzhou, China; 2 Ecology Evolution and Marine Biology, University of California Santa Barbara, Santa Barbara, California, United States of America; University of Zurich, Switzerland

## Abstract

A common hypothesis to explain the effect of litter mixing is based on the difference in litter N content between mixed species. Although many studies have shown that litter of invasive non-native plants typically has higher N content than that of native plants in the communities they invade, there has been surprisingly little study of mixing effects during plant invasions. We address this question in south China where *Mikania micrantha* H.B.K., a non-native vine, with high litter N content, has invaded many forested ecosystems. We were specifically interested in whether this invader accelerated decomposition and how the strength of the litter mixing effect changes with the degree of invasion and over time during litter decomposition. Using litterbags, we evaluated the effect of mixing litter of *M. micrantha* with the litter of 7 native resident plants, at 3 ratios: M_1_ (1∶4, = exotic:native litter), M_2_ (1∶1) and M_3_ (4∶1, = exotic:native litter) over three incubation periods. We compared mixed litter with unmixed litter of the native species to identify if a non-additive effect of mixing litter existed. We found that there were positive significant non-additive effects of litter mixing on both mass loss and nutrient release. These effects changed with native species identity, mixture ratio and decay times. Overall the greatest accelerations of mixture decay and N release tended to be in the highest degree of invasion (mix ratio M_3_) and during the middle and final measured stages of decomposition. Contrary to expectations, the initial difference in litter N did not explain species differences in the effect of mixing but overall it appears that invasion by *M. micrantha* is accelerating the decomposition of native species litter. This effect on a fundamental ecosystem process could contribute to higher rates of nutrient turnover in invaded ecosystems.

## Introduction

In nature, litter of different plant species is typically mixed and the mixtures decompose together. Most studies have shown that mixing different litter species causes litter mixtures to lose mass at different rates than expected from component species incubated in isolation [1], [2], [3]. Non-additive effects are often calculated by comparing observed dynamics of decomposition in litter mixtures with predictions of mass loss, or nutrient concentration change calculated from measured decay rates for each component litter decaying alone [2]. Litter mixing effects may be caused by chemical interactions between the component litters, by changes in the micro-environment in which the litter is decomposed [2]–[5] or by changes in diversity of associated microorganisms and detritivores [6]. Litter mixing studies have focused on detecting these non-additive effects, both in terms of nutrient content of litter [7], [8], [9] and nutrient release from the litter [5], [10]. A review by Gartner & Cardon [2] concluded that mixing of litter often accelerates decomposition with differences between observed and expected mass loss ranging from 1% to 65%. Some studies however, showed that litter mixing can slow decomposition from 1.5% to 22% [2] and indeed a range of results have been found across studies worldwide depending on which litter is mixed together [6].

Biological invasions are recognized as a major driver of altered ecosystem services across the globe. Successful invasive species have potential to influence the nutrient cycling process of the invaded ecosystem, which in turn can affect native plant communities [11], [12]. It has been well documented that litter quality or initial litter chemical components (e.g. N, C/N ratio etc.) are critical factors affecting decomposition rate [13]–[17]. Exotic plant invaders often maintain higher litter N concentrations and lower C:N and tend to decompose more rapidly and release more nitrogen to the soil than native species [12], [18], [19]. They also appear to increase litter decomposition rates over all [20]. It has been proposed that acceleration of decomposition of resident litter as an invader enters a forest may contribute to higher rates of N transformation which in turn could benefit the invader [21].

Liao et al. [20] found that, plant invasion increases litter decomposition rate, probably associated with higher plant nitrogen concentration in the invader compared to natives [18]. Litter of invasive plants is often mixed with litter of native plant species in the invaded ecosystem. Yet most studies of invader versus native litter decomposition are done on unmixed litter [20]. Thus, studying the effect of mixing litter of non-native and native plants may bring new insight to into both our understanding of why litter mixing effects occur, and how ecosystem impacts from invading plants develop. The invading plants could be native or non-native. What we do not understand is how mixing effects change over the time course of an invasion. We address these questions in forest environments in south China where *Mikania micrantha* H.B.K. (thereafter as *M. micrantha*), a non-native vine in the Asteraceae, has abundantly invaded many forests where it alters ecosystem processes [22]. This species is native to tropical Central and South America, is a pest in plantation crops and commercial forests from Mauritius to West Africa and across Asia and it is recognized as one of the top 10 worst weeds in the world [23]. It has been called “mile-a-minute” weed because of its vigorous growth habit [22], [24]. Once established, it is able to kill nearby plants by reducing light and altering soil microbial communities and mineral cycling [22]. While in an invaded mixed community, its stem-leaf biomass ratio (10∶1) is much higher than that in a monotypic *M. micrantha* stand presumably because it allocates more biomass to stems for climbing neighbor plants where they are present [25]. Our previous studies have shown that *M. micrantha* has higher N and lower C than natives and resulted in lower C:N litter relative to natives in invaded sites [26], [27]. Therefore, we predicted there would be positive non-additive effects when litter of *M. micrantha* was combined with native species.

Several studies have demonstrated that the non-additive effects are different during different periods in the decomposition of a litter cohort [3], [28]. This is due to changes in concentrations of water soluble nutrients and structural carbohydrates during different stages of decay [29], [30], [31]. In addition, as an invasion proceeds, the relative amount of the invaders litter changes potentially altering the way in which the mixed pool of litter decays.

In this study, we evaluate the following questions: (1) Do positive non-additive effects occur when litter of *M. micrantha* is mixed with litter of native species? (2) How do the non-additive mixing effects change with different mixing ratios of the litter species? and (3) Do the non-additive mixing effects change over the time course of the litter decomposition process? To test the effects of the mixing *M. micrantha* litter with native litter we compared single-species litter decomposition and C and N concentration for 7 native species with those of mixed litter of *M. micrantha* with each individual native litter type (3 different ratio-mixtures of the native and *M. micrantha*) after 3 decomposition durations (60 days, 128 days, and 180 days decay).

## Materials and Methods

### Ethics Statement

All necessary permits to collect plant material, to treat soil and bury litterbags in the study field were obtained from Dinghushan Forest Experimental Station.

### Litter Collection

We selected 7 resident native evergreen tree species (*Ficus virens*, *Litsea glutinosa*, *Cinnamomum camphora*, *Acacia confusa*, P*inus massoniana*, *Schima superba*, *Castanopsis chinensis*) to study the litter mixing effect with *M. micrantha* based on a study by Zhou et al. [32] from the same site in Guangdong Province. Freshly senesced leaf litter of each resident native species was collected between December 2005 and January 2006. A detailed description of the chemical characteristics of the 7 species litter can be found in Table 1. Litter of *M. micrantha* was mostly vine/stem with few leaves as is typical for this species in a mixed forest setting [25]: in an invaded mixed community, its stem-leaf biomass ratio is 10∶1. *Mikania micrantha* stems were cut as 4–5 cm pieces in order to mix with litter of the 7 native species. The collected litter was air dried and mixed with each species according to treatment. Simultaneously, subsamples were oven dried and ground into powder using a basic analytical mill (IKA, Germany). The initial litter concentrations of C and N were then determined by dry combustion on a CHNS analyzer (Vario El, Germany).

**Table 1:** Original Carbon and nitrogen content of plant litter.

Species	Family	C	N	C/N
*Ficus virens*	Moraceae	41.34	1.46	28.26
*Litsea glutinosa*	Lauraceae	48.65	1.56	31.13
*Cinnamomum camphora*	Lauraceae	49.52	1.16	42.54
*Acacia confusa*	Fabaceae	49.94	1.14	43.69
*Pinus massoniana*	Pinaceae	49.58	0.88	56.34
*Schima superba*	Theaceae	49.84	1.08	46.15
*Castanopsis chinensis*	Fagaceae	48.42	1.03	47.10
* *Mikania micrantha H.B.K.*	Asteraceae	36.03	1.79	20.14

* non-native invasive species in Guangdong,southern China; Litter of the invasive species *Mikania micrantha* includes stems and leaves, while litter of above 7 natives are only leaves.

### Experimental Setup

We used a buried litterbag technique to quantify litter decomposition. Single-species litterbags were used to generate decomposition rates and N and C release for pure litter. These rates were used as a baseline for comparison with mixed-species litter-bags to determine if interaction effects occurred when litter of different species was mixed. In order to measure the mixture effect on litter decomposition and nutrient release at different degrees of *M. micrantha* invasion, litter of *M. micrantha* was mixed with the above 7 native plants respectively at the following ratios: M_1_ (1∶4, exotic:native), M_2_ (1∶1) and M_3_ (4∶1). These three ratios are hereafter referred to as the low (M_1)_, medium (M_2)_, and high (M_3_ )degrees of invasion. Total litter mass/bag was 10 g with 12 replicate bags per species and per species mixture. There were 4 replicate bags for each combination of mixture and sampling date.

To reduce the effect of habitat variability on decomposition dynamics, we selected a flat open area in a subtropical forest area of Dinghushan Forest Experimental Station (N23°10', E112°10'), southern China, where the annual relative humidity is 80%, the mean annual temperature is 21°C, and the mean annual rainfall is 1927 mm. The litter layer in the forest was moved aside and the top 20 cm of soil mixed to create a homogenous environment across the area where the bags were to be set out. Nylon-net bags (15 cm×20 cm, 1 mm mesh) containing air dried litter were buried horizontally at a shallow depth (approx. 5 cm) as others have done [33], [34], [35]. All the litterbags were buried at the beginning of April 2006 with a randomized block design. Four litterbag of each type were retrieved after 60 d, 128 d, 180 d decay. These were brushed lightly to remove soil and roots, rinsed, dried at 60°C, and dry mass determined. The litter was ground and analyzed for C and N content as described above. Mass loss and N concentration data were used to calculate changes in the absolute amount of N (net immobilization or release).

### Calculations

Observed litter mass loss (%) of both single-species and mixed-species was calculated as below:

Observed litter mass loss (%) = (M*_lb_*–M*_la_*)/M*_lb_* × 100 (1).

Where, Mlb: Litter mass in the litterbags before decomposition, Mla: Litter mass remaining after decomposition.

To determine whether interactions in litter mixtures occurred, we compared the predicted mass loss, using the mass loss from the single-species litterbags of the component species adjusted for proportional weight in the mix to the actual value observed for mixture. This was calculated as follows [1], [8] where PLML stands for Predicted Litter Mass Loss:

PLML % = [*M*_nat_/(*M*_nat_+*M_non_*) × *LML*_nat_+*M_non_*/(*M*_nat_+*M*_non_) × *LML*_non_] (2).

In this equation, *LML*_nat_ and *LML*_non_ are the litter mass loss (%) of single-species litterbag of native species and non-native species respectively, and *M*_nat_ and *M*_non_ are the initial litter dry mass of these species in the mixture.

The strength of the mixed litter decomposition interaction is defined as [8]:

 = 1– (observed/predicted remaining mass) (3).

In the absence of a mixture interaction the value of these parameters should be (close to) zero. As we used the remaining mass in the calculations, positive and negative interactions would yield values that are respectively greater or smaller than zero. A positive value indicates facilitation (faster decomposition observed than predicted value), while a negative value indicates inhibition. Stronger mixture interactive effects would lead to a greater departure from zero (either positive or negative).

Mixing strength values for carbon and for nitrogen were calculated in a similar manner to the mixing strength values for mass loss. The details are shown in Appendix S1.

### Statistical Analysis

The litter mass loss for the 8 individual species calculated for each of the collection times (60, 128 and 180 days). Litter mass loss of single species over each of the 3 decay times was tested with one-way ANOVA at P<0.05. For each mixture we tested whether the interactions (mixing effects) differed significantly from zero using one sample t-tests, and a significance value of *P*<0.01 (indicated by ** on graphs), *P*<0.05 (*) and *P*<0.10 (#). In order to know the differences in mixing interaction strength between litter types (species), decay ratio over time, as well as the interactions between the factors, we used mixed effect model with mixture type (species) and mixing ratio as fixed effects and decay time as a random effect. All analyses were performed using SPSS 18.0 for Windows (SPSS, Chicago, Illinois, USA).

## Results

### Single-species Litter Mass Loss

**Figure 1:**
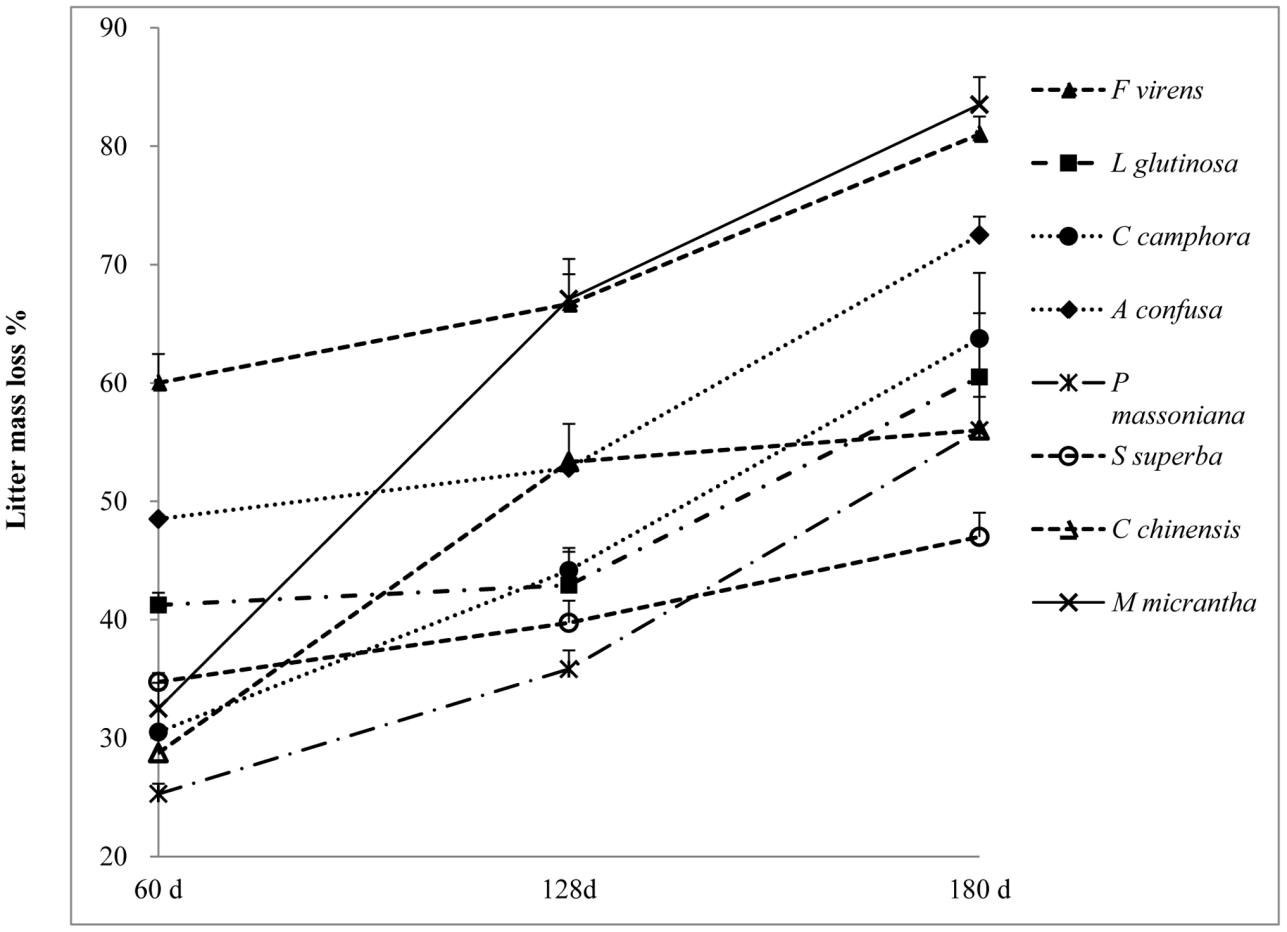
Mean (+SE, n = 4) percentage of litter mass loss of the 7 native single species and non-native (exotic) invasive species *M. micrantha* after 60 d, 128 d and 180 d decay. The analysis of litter mass loss among 8 single species over each decay time by ANOVA is shown in Table S1.

There were significant differences in single-species litter mass loss among the 8 species (Figure 1, Table S1). Using average decay times across the three intervals, the native species *Ficus virens* had the greatest litter mass loss, *M. micrantha* was the second, followed by *A. confusa, L. glutinosa, C. camphora, C. chinensis, S. superba,* and the lowest was *P. massoniana*.

### Mixing Effect on Litter Mass Loss

**Figure 2:**
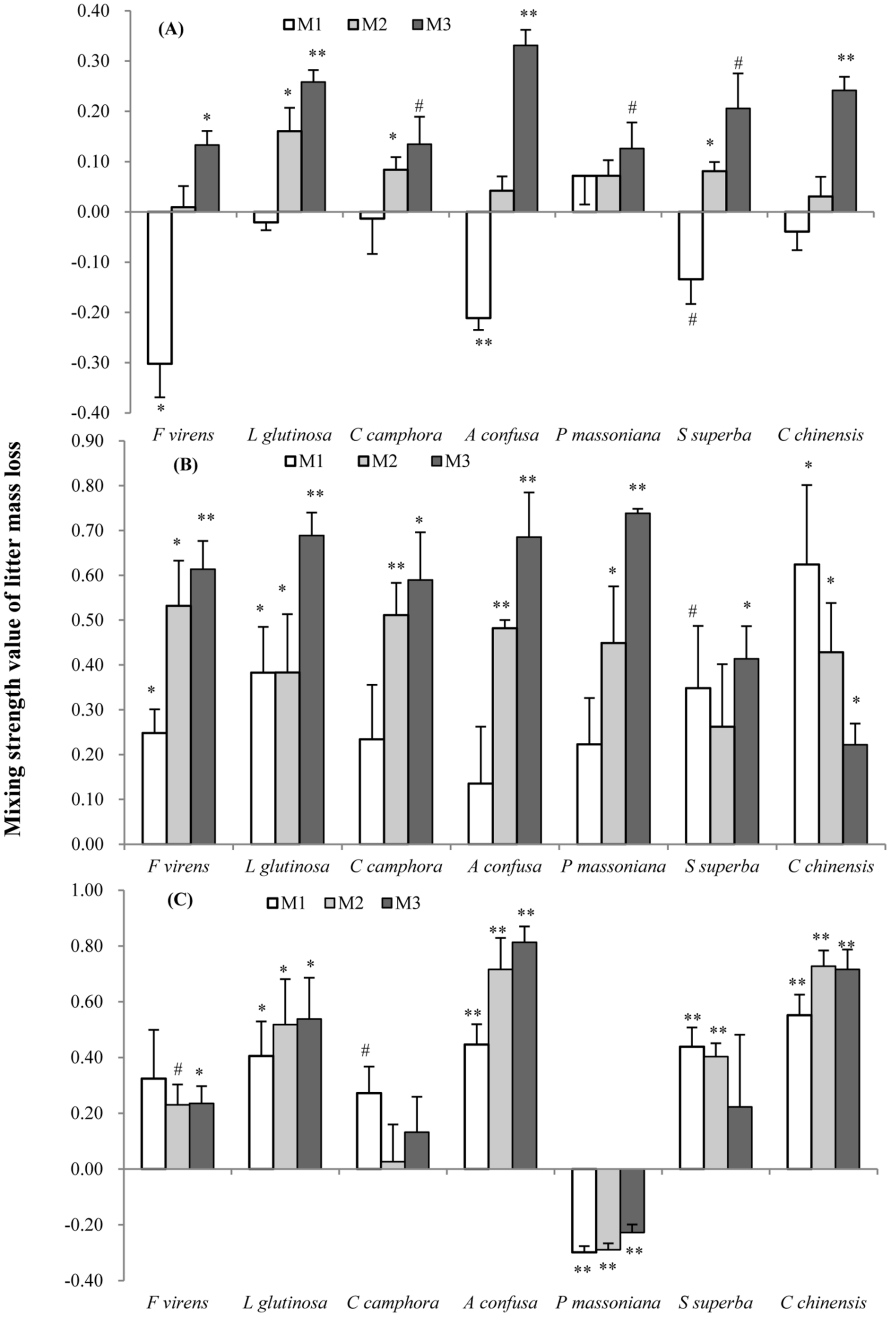
Mean mixing interaction strength values (+SE, n = 4) of litter mass loss after 60 (A), 128 (B) and 180 (C) days decay. M_1_ (1∶4, mixture with 20% exotic litter), M_2_ (1∶1) and M_3_ (4∶1, mixture with 80% exotic litter) represent 3 different litter mixing ratio of *M. micrantha* to native species. Non-additive interactions are significantly different from zero with (**) at P<0.01, (*) at P<0.05, and with (#) at P<0.10 tested separately with one sample t test. See Table S4 for observed litter mass loss.

The results showed that there are significant non-additive mixing effects on litter mass loss with all mixture species and under the 3 mixing ratios. All species responded similarly to mixing ratio (species×ratio interaction P = 0.280, *F_12,229_* = 1.206, Table 2, Figure 2). Most non-additive effects of mixing ratio M_1_ (low degree of invasion) are negative during the first 60 days decay (Figure 2A), but after 128 and 180 days, most are positive except *P. massoniana*. *P. massoniana* behaved similarly to the other species at the early and middle stages of decomposition but showed inhibition of decomposition in the mixture after 180 days decay (Figures 2B and 2C). Non-additive effects were largely positive for all species in all decay periods in the medium and higher invasion mixtures (Figure 2A). These two higher ratios of invasion (M_2_ & M_3_) did not differ in their degree of non-additivity.

**Table 2:** Analyses of litter mixing effects for litter mass loss and nutrient release were performed by mixed effect model with mixture type (species) and mixing ratio as fixed effects and decay time as a random effect.

Source	NumDF	DenDF	Litter mass loss		Litter N release			Litter C release		
			F	P	F	P	F		P	
Species	6	229	7.354	0.000	6.364	0.000	5.563		0.000	
Ratio	2	229	13.574	0.000	3.787	0.024	1.021		0.362	
Species × Ratio	12	229	1.206	0.280	1.013	0.437	1.899		0.035	

### Mixing Effect on Litter N and C Release

**Figure 3:**
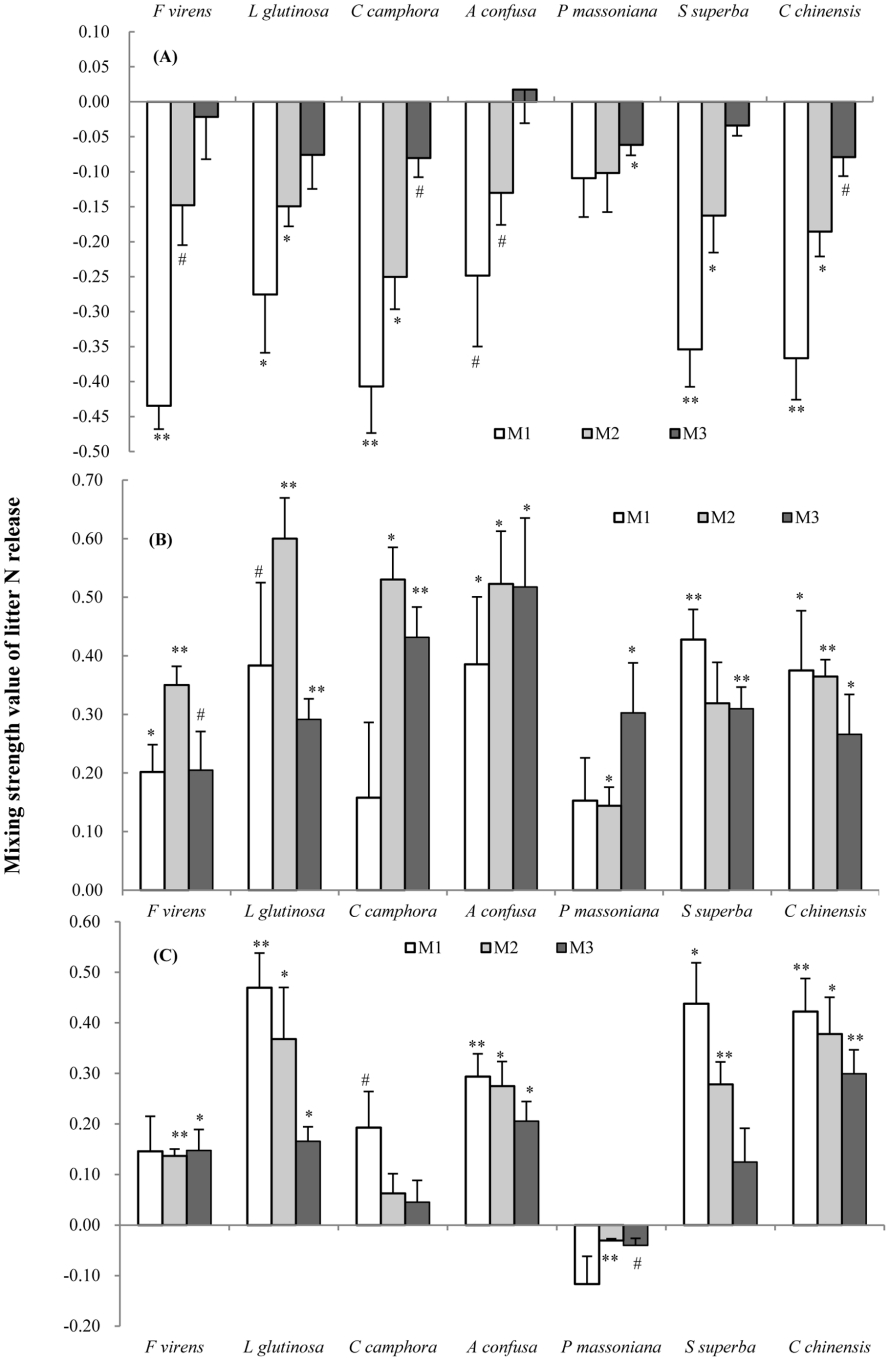
Mean mixing interaction strength values (+SE, n = 4) of litter N releases after 60 (A), 128 (B) and 180 (C) days decay. M_1_ (1∶4, mixture with 20% exotic litter), M_2_ (1∶1) and M_3_ (4∶1, mixture with 80% exotic litter) represent 3 different litter mixing ratio of *M. micrantha* to native species. Non-additive interactions are significantly different from zero with (**) at P<0.01, (*) at P<0.05, and with (#) at P<0.10 tested separately with one sample t test. See Table S5 for observed N release.

As with litter mass loss, most non-additive effects from litter mixing are negative during the first 60 days decay (Figure 3A), that is, N release in mixture was delayed compared to the species alone. However, after 128 and 180 days of decay, most effects are positive although *P*. *massoniana* again showed negative effects after 180 days decay (Figures 3B and 3C). The mixing effect on N release varied with species and mixture ratio but the species × ratio interaction was not significant for N release (Table 2, Figure 3). The acceleration of litter N decay by mixing (any ratio) was strongest during the second decay period (Figure 3).

**Figure 4:**
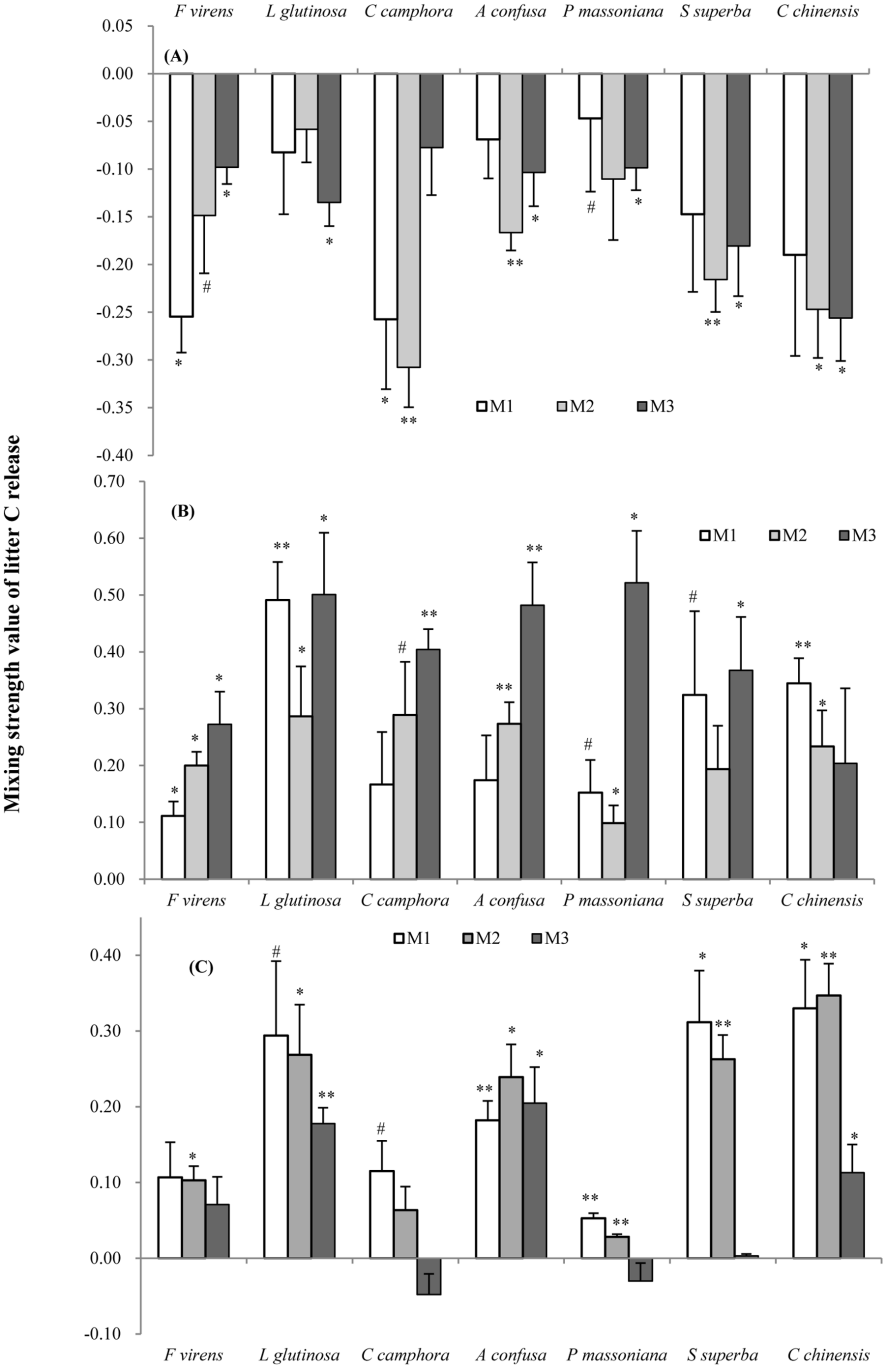
Mean mixing interaction strength values (+SE, n = 4) of litter C releases after 60 (A), 128 (B) and 180 (C) days decay. M_1_ (1∶4, mixture with 20% exotic litter), M_2_ (1∶1) and M_3_ (4∶1, mixture with 80% exotic litter) represent 3 different litter mixing ratio of *M. micrantha* to native species. Non-additive interactions are significantly different from zero with (**) at P<0.01, (*) at P<0.05, and with (#) at P<0.10 tested separately with one sample t test. See Table S6 for observed C release.

As with N release, there were significant non-additive effects of litter mixing on C release in all of the 7 mixture species, but there was no consistent effect of the mixing ratios nor did native species identity affect whether mixing ratio was significant (Table 2). As with N, most non-additive effects of litter mixing were negative during the first 60 days decay (Figure 4A), but during the second and third decay periods C release was accelerated in all mixtures with strong net positive effects in the second period that declined somewhat during the third period (Figures 4B and 4C).

### Relationship between Mixing Effect and Initial Litter N Content

Contrary to predictions, there were no significant correlations between the strength of the mixing effect on litter mass loss, N release or C release and difference in initial single litter N content between the native species and *M. micrantha*. *Mikania* was higher in N and lower in C/N of all residents. Likewise there were no significant correlations between the strength of the mixing effect and the difference in C/N ratio of residents versus *M. micrantha* at any of the 3 collection dates for any of the measured parameters (Table S2).

### Overall Results of Non-additive Effects

In order to understand the general results of mixing on litter mass loss, N release and C release among species the average value of litter mixing interaction strength was calculated for each species by averaging across ratios and times. Similarly, to compare mixing ratios with each other, the average value of litter mixing interaction strength of each mixing ratio was calculated by averaging across all mixture types and decay times. To evaluate the importance of decay time, the average value was calculated across all mixture types and mixing ratios. Among the 3 mixing ratios, the mixing effects on litter mass loss and C release were the highest under M_3_ (high degree of invasion), while the strongest effect on litter N release was under M_2_ (medium degree of invasion) (Table S3). Across the different decay times, the non-additive mixing effects were consistently the highest in the middle decay period (128 days, Table S3).

## Discussion

### Non-additive Effect of Litter Mixing on Litter Mass Loss and Nutrient Release

Our experiment was designed to provide insight into the outcome of forest invasion by *M. micrantha* in terms of decomposition of native species leaf litter. We found that 63.5% of litter mixtures showed non-additive effects on litter mass loss (marked with star in Figure 2). Among the mixtures with non-additive effects, 87.5% were positive for litter mass loss demonstrating that invasion is accelerating litter decomposition of the native species (Figure 2). Acceleration was seen even at the lowest level of invasion measured (20% exotic litter) for most species by middle and final decay periods.

A review by Gartner & Cardon [2] concluded that non-additive effects were more common than not with 66.7% of 162 mixtures showing non-additive effects on litter mass loss. The observed mass loss in some mixtures was as much as 65% more rapid than expected from single-species litter, but more often mass loss in mixtures exceeded expected decay by ≤20%. Gartner & Cardon [2] also found that nutrient transfer among leaves of different species is striking, with 76% of 123 mixtures showing non-additive dynamics of nutrient concentrations. In our present study, the average mixing effect on litter mass loss was 28%, consistent with the Gartner & Cardon findings [2] and the greatest non-additive effect on litter mass loss was 0.81 (Figure 2C-species *A. confusa*-ratio M_3_). Interestingly, this was an N-fixing tree in a genus shown elsewhere to contribute to enhanced soil N cycling [36], [37].

It has been shown that mesh size of litterbags can influence decay processes by altering entry of soil fauna [38]. In the present study, 1 mm mesh size was used, a size which excludes large macro-detritivores which selectively can feed on one of the species in a mixture. This potentially may alter the outcome of interactions between litter species in a mixture possibly by slowing decomposition. During the harvest of litterbags, we observed that there were more earthworms surrounding the litterbags containing litter of *M. micrantha* than those bags without *M. micrantha* litter (e.g. native single-species litterbags). Other studies of invading plants have found that earthworm densities are enhanced beneath some N rich species [39], [40]. A meta-analysis of effects of litter diversity (mixing) versus consumer (detritivore) diversity on overall decomposition rates demonstrated that detritivore diversity plays a more important role in decomposition rates than does litter diversity [41]. We did not quantify differences in the microbial or invertebrate communities between our litter mixtures but the relatively consistent accelerated decomposition in our mixed litterbags suggests that even without macro-detritivores, this invasion accelerates decomposition of native species. Gessner et al. [6] review evidence that substrate diversity promotes microbial diversity during decomposition and Ashton et al. [19] suggest that increased substrate diversity as during invasion could enhance microbial efficiency during decomposition. Also in this system, enhanced substrate diversity, could promote either increased detritivore diversity or enhanced detritivore activity (e.g. earthworm foraging) either of which could accelerate decomposition [42], [43]. In natural conditions, the non-additive effects of this invasion on litter decomposition are likely even greater than we measured since no mesh would be present to block macro-detritivores (e.g. earthworm).

### Litter Mixing Effect and Initial Litter N Content

We found that the plant species used in the litter mixture significantly influenced the strength and direction of the non-additive effects (Table 2). One hypothesis explaining the effect of litter mixing is based on the difference of litter N content between the mixed species [4], [44]. Some studies have suggested that litter mixture effects might be greater when the component species differ greatly in their litter nutrient concentration [45], [46]. Wardle et al. [45] for example, found that when litter of plants with high nitrogen status were mixed, synergistic interactions between species causing enhanced decomposition were more likely. Our data however, do not support the hypothesis that the non-additive effect of mixing can be predicted by initial differences in %N (Table S2). Thus factors other than N are likely to be important. For example, we found that the biggest difference in N content between *M. micrantha* and a native litter type was with *P. massoniana* (Table 1). Yet the mixing effect of *P. massoniana* with *M. micrantha*, was not the strongest non-additive effect in litter mass loss and nutrient release and was often negative (Table S3). We believe that this is due to polyphenolic compounds found in litter of *P. massoniana* [47] and that these might be slowing the decomposition process in the entire mix. It has been reported that polyphenols complex with proteins in leaf material forming polyphenol-protein complexes, which are resistant to most decomposing organisms, and consequently slow down decomposition [48], [49].

Overall our results showed there was no significant correlation between the mixing effect on litter mass loss, N release and C release and the difference in initial single litter N content between the mixed species or the difference in their C/N ratio over the 3 decomposition times (Table S2). Other studies have also found that interactions in litter mixtures are unrelated to differences in litter chemistry between the mixed species [8]. Although nutrient transfer was not measured here and there was no significant correlation between mixing effect and litter initial components, nutrient transfer could have occurred. *Mikania micrantha* is higher in N content than any of the natives (Table 1) so N transfer could be important. Nutrient transfer might be influenced by P (often important for microbial driven decomposition), Mn or other elements in the litter [31] which we did not measure. In addition, physical conditions (e.g. soil water content) and litter physical traits (e.g. water hold capacity) might result in non-additive effects [50], [51]. In the present study, soil water content was measured when we harvested the litterbags, and the results showed that there is no significant difference in soil water content around the various litterbags (data not shown) and moisture content was high overall due to the plentiful rainfall (mean annual precipitation 1927 mm) in this subtropical area in southern China. This suggests that soil water content is unlikely to be an important factor contributing to the non-additive effects measured.

### Litter Mixture Ratio and Temporal Effects

Past researchers have generally used mixtures containing equal masses of each component litter type [2], with a few exceptions [52], [53], [54]. Only a few studies evaluated the importance of an invader on decomposition of existing native tree leaf litter using a mixture ratio typical of what was found on the forest floor of an invaded site [52], [53]. The present experiment was designed using 3 mixing ratios, which permitted us to determine the magnitude of potential effects of litter mixing as when an invader becomes abundant in an ecosystem.

Changes in species relative abundance of leaf litter mixtures can affect mixture decomposition [55], [56]. In the present study, the non-additive effects on litter mass loss were highest under M_3_ (exotic litter dominant). Yet mixtures containing equal masses of each species (M_2_) showed the strongest interaction strength for N release. It has been well documented that higher N content and lower C-N ratio litter will decompose faster [15]. Mixtures containing more litter of *M. micrantha* with higher N and lower C-N ratio should thus accelerate the litter mass loss of the entire litter mixture as we observed. However, for N release it is unclear why the acceleration of N release was greatest in M_2_ but it suggests that trait evenness may be important in generating non-additive effects. It is also possible that after the invader comes to dominate a site such as we mimicked in our M_3_ treatment, microbial and detritivore activity may decline as the native inputs decline.

Our study is consistent with other studies that report that the litter mixing effects varies over the course of decay [3], [50]. Non-additive effects were typically negative in the initial decay period, a time period in decomposition when immobilization of nutrients can be high [31]. Non-additive effects reached their highest positive values during the middle period (with the exception of *P. massoniana*, Figures 2, 3, 4). This delay could be due to both the immobilization of nutrients early on and the time needed for detritivores to accumulate or for microbial diversity to track substrate diversity [6]. The empirical relationship between detritivore diversity, litter diversity and time has been poorly studied in terrestrial ecosystems [6].

### Implications for Invasion

To manage non-native invasive species or restore invaded ecosystems, it is necessary to understand the mechanisms through which invasive plant species may alter an invaded ecosystem. Most previous studies of the impacts of invasive plant on the invaded ecosystem have focused on their effects on litter production, chemistry and single species decay rates [12], [20], [57], [58]. However, evaluation of the dependence of decomposition on the mixing ratio of invader litter with residents provides insight into understanding the time course of changes in nutrient cycling following invasion. In a litter mixture of invasive and native species, decomposer organisms may preferentially exploit a higher quality invasive litter, allowing nutrient transfer to the lower quality native litter, leading to a more rapid, synergistic decomposition of the entire mixture [4] a dynamic we observed. If *M. micrantha* depends on high N availability to maintain high growth rates, then acceleration of litter decomposition as it invades could ultimately aid in sustaining these growth rates. Our data suggest that the greatest acceleration of mixture decay and N release tended to be in the higher degrees of invasion (M_2_ & M_3_)(Tables S4, S5 and S6). If this is a positive feedback to *M. micrantha* success then this feedback appears to increase as invasion progresses. *Mikania micrantha* invasion into forest patches can occur very rapidly [22], [59] and it may be that the accelerated breakdown of native forest litter in the presence of this invader, contributes to the facilitation of this invasion. Few studies document longterm impacts of invaders [60]. Yet as native species decline in an ecosystem, litter diversity and associated organisms should also decline which could further influence decompositional dynamics.

## Supporting Information

Table S1 **The analyses of litter mass loss among 8 single species over each decay time by ANOVA.**
(DOCX)

Table S2 **Pearson coefficients between mixing effect of litter mass loss, N, C release with the difference in initial single litter N content and C/N ratio.**
(DOCX)

Table S3 **Overall results of mixing effect on litter mass loss, N release and C release between litter mixing types, between mixing ratios, between decay time.**
(DOCX)

Table S4 **Observed litter mass loss.**
(DOCX)

Table S5 **Observed litter N release.**
(DOCX)

Table S6 **Observed litter C release.**
(DOCX)

Appendix S1 **Methods of calculating N and C release.**
(DOCX)
