# Supplementary material for: Non-Additive Effects on Decomposition from Mixing Litter of the Invasive Mikania micrantha H.B.K. with Native Plants
Source: PLoS One. 2013 Jun 20;8(6):e66289. doi: 10.1371/journal.pone.0066289 (PMC3688783; doi:10.1371/journal.pone.0066289)
Supplement: Table S1 — The analyses of litter mass loss among 8 single species over each decay time by ANOVA. (DOCX) [file pone.0066289.s001.docx]

| **Table S1.** The analyses of litter mass loss among 8 single species over each decay time by two-way ANOVA.   \| Source \| Sum Squares \| df \| Mean Square \| F \| Sig. \| Partial Eta Squared \| \| --- \| --- \| --- \| --- \| --- \| --- \| --- \| \| species \| 9617.838 \| 7 \| 1373.977 \| 37.897 \| 0.000 \| 0.787 \| \| Time \| 12018.855 \| 2 \| 6009.428 \| 165.754 \| 0.000 \| 0.822 \| \| species * Time \| 2832.029 \| 14 \| 202.288 \| 5.580 \| 0.000 \| 0.520 \| \| Error \| 2610.372 \| 72 \| 36.255 \|  \|  \|  \| \| Total \| 277171.011 \| 96 \|  \|  \|  \|  \| |
| --- | --- | --- | --- | --- | --- | --- | --- | --- | --- | --- | --- | --- | --- | --- | --- | --- | --- | --- | --- | --- | --- | --- | --- | --- | --- | --- | --- | --- | --- | --- | --- | --- | --- | --- | --- | --- | --- | --- | --- | --- | --- | --- |
