# Supplementary material for: Non-Additive Effects on Decomposition from Mixing Litter of the Invasive Mikania micrantha H.B.K. with Native Plants
Source: PLoS One. 2013 Jun 20;8(6):e66289. doi: 10.1371/journal.pone.0066289 (PMC3688783; doi:10.1371/journal.pone.0066289)
Supplement: Table S2 — Pearson coefficients between mixing effect of litter mass loss, N, C release with the difference in initial single litter N content and C/N ratio. (DOCX) [file pone.0066289.s002.docx]

| **Table S2.** Pearson coefficients between mixing effect of litter mass loss, N, C release with the difference in initial single litter N content and C/N ratio. Here we only analyzed the coefficients under the mixture ratio M_2_ (2 equally mixed litter) to understand if litter chemistry do explain the mixing effect. The difference in their initial N content and C/N ratio equals litter N content of species-A minus species-B divided by species A.   \|  \| **Mixing effect on litter mass loss** \| \| \| \| \|  \| **Mixing effect on litter N release** \| \| \| \| \|  \| **Mixing effect on litter C release** \| \| \| \| \| \| \| --- \| --- \| --- \| --- \| --- \| --- \| --- \| --- \| --- \| --- \| --- \| --- \| --- \| --- \| --- \| --- \| --- \| --- \| --- \| \| **60 d** \| **128 d** \| \| **180 d** \| \|  \| **60 d** \| \| **128 d** \| \| **180 d** \|  \| **60 d** \| **128 d** \| \| \| **180 d** \| \| \| **N difference** \| -0.314 \| \| -0.137 \| \| -0.284 \|  \| 0.090 \| -0.643 \| \| -0.359 \| \|  \| -0.410 \| \| -0.501 \| -0.130 \| \| \| ***P* value** \| 0.493 \| \| 0.769 \| \| 0.537 \|  \| 0.847 \| 0.119 \| \| 0.429 \| \|  \| 0.361 \| \| 0.252 \| 0.781 \| \| \| **C/N difference** \| -0.105 \| \| -0.223 \| \| -0.315 \|  \| 0.167 \| -0.597 \| \| -0.338 \| \|  \| -0.267 \| \| -0.494 \| -0.118 \| \| \| \| ***P* value** \| 0.822 \| \| 0.630 \| \| 0.492 \|  \| 0.721 \| 0.157 \| \| 0.458 \| \|  \| 0.562 \| \| 0.260 \| 0.802 \| \| \| |
| --- | --- | --- | --- | --- | --- | --- | --- | --- | --- | --- | --- | --- | --- | --- | --- | --- | --- | --- | --- | --- | --- | --- | --- | --- | --- | --- | --- | --- | --- | --- | --- | --- | --- | --- | --- | --- | --- | --- | --- | --- | --- | --- | --- | --- | --- | --- | --- | --- | --- | --- | --- | --- | --- | --- | --- | --- | --- | --- | --- | --- | --- | --- | --- | --- | --- | --- | --- | --- | --- | --- | --- | --- | --- | --- | --- | --- | --- | --- | --- | --- | --- | --- | --- | --- | --- | --- | --- | --- | --- | --- | --- | --- | --- | --- | --- | --- | --- | --- | --- | --- | --- | --- | --- | --- | --- | --- | --- | --- | --- | --- | --- |
