# Supplementary material for: Non-Additive Effects on Decomposition from Mixing Litter of the Invasive Mikania micrantha H.B.K. with Native Plants
Source: PLoS One. 2013 Jun 20;8(6):e66289. doi: 10.1371/journal.pone.0066289 (PMC3688783; doi:10.1371/journal.pone.0066289)
Supplement: Table S3 — Overall results of mixing effect on litter mass loss, N release and C release between litter mixing types, between mixing ratios, between decay time. (DOCX) [file pone.0066289.s003.docx]

**Table S3.** Overall results of mixing effect on litter mass loss, N release and C release between litter mixing types, between mixing ratios, between decay time. Data are the average of litter mixing interaction strength based on the average of each litter mixture type (species) regardless of the ratio and time. Similarly, between mixing ratio, the average of litter mixing interaction strength of each mixing ratio was calculated regardless of mixture type and decay time. Between decay time, the average was calculated regardless of mixture type and mixing ratio.

|  | Litter mixing types (native species with exotic invasive *M*. *micrantha*) | | | | | | |  | Litter mixing ratio | | |  | Decay time | | |
| --- | --- | --- | --- | --- | --- | --- | --- | --- | --- | --- | --- | --- | --- | --- | --- |
| Mixing effect | *F. virens* | *L. glutinosa* | *C. camphora* | *A. confusa* | *P. massoniana* | *S. superba* | *C. chinensis* |  | M_1_ | M_2_ | M_3_ |  | 60 d | 128 d | 180 d |
| Mass loss | 0.229 | 0.382 | 0.222 | 0.381 | 0.091 | 0.257 | 0.382 |  | 0.175 | 0.286 | 0.371 |  | 0.059 | 0.442 | 0.332 |
| N release | 0.065 | 0.198 | 0.076 | 0.204 | 0.016 | 0.149 | 0.164 |  | 0.083 | 0.151 | 0.140 |  | -0.174 | 0.345 | 0.203 |
| C release | 0.041 | 0.194 | 0.039 | 0.135 | 0.049 | 0.103 | 0.097 |  | 0.094 | 0.078 | 0.110 |  | -0.161 | 0.290 | 0.152 |
