# Supplementary material for: Non-Additive Effects on Decomposition from Mixing Litter of the Invasive Mikania micrantha H.B.K. with Native Plants
Source: PLoS One. 2013 Jun 20;8(6):e66289. doi: 10.1371/journal.pone.0066289 (PMC3688783; doi:10.1371/journal.pone.0066289)
Supplement: Table S4 — Observed litter mass loss. (DOCX) [file pone.0066289.s004.docx]

**Table S4** Observed litter mass loss (%) (Value ± SD) after 60, 128 and 180 days decomposition when they were mixed with non-native invasive plant *M*. *micrantha* under 3 different mixing proportions (M_1_, M_2_ and M_3_). M_1_, *M*. *micrantha* : Native = 1:4; M_2_, *M*. *micrantha* : Native = 1:1; M_3_, *M*. *micrantha* : Native = 4:1.

| Native species | | 60 days | | | | |  | 128 days | | | | |  | 180 days | | | | |
| --- | --- | --- | --- | --- | --- | --- | --- | --- | --- | --- | --- | --- | --- | --- | --- | --- | --- | --- |
|  |  | **M_1_** | **M_2_** | | **M_3_** | |  | **M_1_** | | **M_2_** | | **M_3_** |  | **M_1_** | **M_2_** | | **M_3_** | |
| *F. virens* | 40.75± 3.30 | | | 46.75±3.77 | | 46.25±4.03 |  | 75.00±12.36 | 84.50±8.06 | | 87.25±5.12 | |  | 87.50±8.23 | | 86.33±3.79 | | 87.00±12.00 |
| *L. glutinosa* | 38.25±3.40 | | | 49.00±3.94 | | 51.25±2.99 |  | 67.75±7.97 | 72.25±17.17 | | 88.25±3.59 | |  | 79.25±16.68 | | 86.50±9.00 | | 90.25±6.40 |
| *C. camphora* | 30.00±3.16 | | | 37.25±2.87 | | 38.75±7.64 |  | 60.75±21.08 | 67.50±6.86 | | 83.50±10.34 | |  | 76.50±6.95 | | 74.33±15.31 | | 82.25±6.40 |
| *A. confusa* | 33.75±2.75 | | | 43.00 ±3.37 | | 54.25±4.58 |  | 61.67±15.31 | 79.25±1.50 | | 88.75±7.14 | |  | 86.00±3.16 | | 93.75±4.57 | | 96.50±2.08 |
| *P. massoniana* | 32.00±7.83 | | | 34.00±3.83 | | 39.75±6.99 |  | 50.00±2.94 | 61.00±5.66 | | 73.00±2.00 | |  | 55.00±14.58 | | 73.25±13.40 | | 89.75±0.50 |
| *S. superba* | 25.50 ±7.33 | | | 39.00±2.83 | | 46.75 ±9.18 |  | 61.33±24.42 | 62.50±17.46 | | 76.50±5.69 | |  | 74.33±9.61 | | 79.25±3.10 | | 81.50±13.53 |
| *C. chinensis* | 26.75±3.30 | | | 32.75±5.32 | | 48.25±3.10 |  | 73.50±11.70 | 77.25±8.62 | | 72.25±9.78 | |  | 82.75±3.95 | | 91.75±3.30 | | 93.75±4.79 |
