# Supplementary material for: Non-Additive Effects on Decomposition from Mixing Litter of the Invasive Mikania micrantha H.B.K. with Native Plants
Source: PLoS One. 2013 Jun 20;8(6):e66289. doi: 10.1371/journal.pone.0066289 (PMC3688783; doi:10.1371/journal.pone.0066289)
Supplement: Table S5 — Observed litter N release. (DOCX) [file pone.0066289.s005.docx]

**Table S5** Observed litter N release (g N per litter bag ) (Value ± SD) after 60, 128 and 180 days decomposition when they were mixed with non-native invasive plant *M*. *micrantha* under 3 different mixing proportions (M_1_, M_2_ and M_3_). M_1_, *M*. *micrantha* : Native = 1:4; M_2_, *M*. *micrantha* : Native = 1:1; M_3_, *M*. *micrantha* : Native = 4:1.

| Native species | | 60 days | | | | |  | 128 days | | | | |  | 180 days | | | | |
| --- | --- | --- | --- | --- | --- | --- | --- | --- | --- | --- | --- | --- | --- | --- | --- | --- | --- | --- |
|  |  | **M_1_** | **M_2_** | | **M_3_** | |  | **M_1_** | | **M_2_** | | **M_3_** |  | **M_1_** | **M_2_** | | **M_3_** | |
| *F. virens* | 0.052± 0.001 | | | 0.081±0.010 | | 0.105±0.008 |  | 0.113±0.012 | 0.141±0.010 | | 0.153±0.070 | |  | 0.132±0.014 | | 0.146±0.005 | | 0.161±0.014 |
| *L. glutinosa* | 0.033±0.006 | | | 0.061±0.006 | | 0.090±0.006 |  | 0.090±0.011 | 0.108±0.038 | | 0.154±0.007 | |  | 0.109±0.014 | | 0.141±0.018 | | 0.154±0.013 |
| *C. camphora* | 0.030±0.003 | | | 0.054±0.007 | | 0.089±0.003 |  | 0.068±0.045 | 0.108±0.011 | | 0.136±0.002 | |  | 0.087±0.012 | | 0.109±0.021 | | 0.137±0.011 |
| *A. confusa* | 0.035±0.010 | | | 0.063±0.008 | | 0.099±0.006 |  | 0.073±0.019 | 0.119±0.008 | | 0.151±0.009 | |  | 0.106±0.005 | | 0.137±0.007 | | 0.151±0.003 |
| *P. massoniana* | 0.041±0.014 | | | 0.063±0.007 | | 0.097±0.011 |  | 0.050±0.012 | 0.086±0.001 | | 0.149±0.047 | |  | 0.050±0.002 | | 0.089±0.012 | | 0.118±0.016 |
| *S. superba* | 0.033 ±0.015 | | | 0.059±0.008 | | 0.091±0.007 |  | 0.064±0.007 | 0.095±0.027 | | 0.134±0.016 | |  | 0.085±0.013 | | 0.116±0.004 | | 0.138±0.019 |
| *C. chinensis* | 0.034±0.017 | | | 0.057±0.010 | | 0.089±0.006 |  | 0.097±0.017 | 0.113±0.011 | | 0.130±0.026 | |  | 0.092±0.005 | | 0.130±0.004 | | 0.156±0.006 |
