# Supplementary material for: Non-Additive Effects on Decomposition from Mixing Litter of the Invasive Mikania micrantha H.B.K. with Native Plants
Source: PLoS One. 2013 Jun 20;8(6):e66289. doi: 10.1371/journal.pone.0066289 (PMC3688783; doi:10.1371/journal.pone.0066289)
Supplement: Table S6 — Observed litter C release. (DOCX) [file pone.0066289.s006.docx]

**Table S6** Observed litter C release (g C per litter bag ) (Value ± SD) after 60, 128 and 180 days decomposition when they were mixed with non-native invasive plant *M*. *micrantha* under 3 different mixing proportions (M_1_, M_2_ and M_3_). M_1_, *M*. *micrantha* : Native = 1:4; M_2_, *M*. *micrantha* : Native = 1:1; M_3_, *M*. *micrantha* : Native = 4:1.

| Native species | | 60 days | | | | |  | 128 days | | | | |  | 180 days | | | | |
| --- | --- | --- | --- | --- | --- | --- | --- | --- | --- | --- | --- | --- | --- | --- | --- | --- | --- | --- |
|  |  | **M_1_** | **M_2_** | | **M_3_** | |  | **M_1_** | | **M_2_** | | **M_3_** |  | **M_1_** | **M_2_** | | **M_3_** | |
| *F. virens* | 1.962± 0.108 | | | 2.073±0.261 | | 2.170±0.141 |  | 3.283±0.240 | 3.384±0.148 | | 3.074±0.132 | |  | 3.663±0.240 | | 3.498±0.103 | | 3.255±0.419 |
| *L. glutinosa* | 1.970±0.247 | | | 2.007±0.294 | | 1.820±0.071 |  | 3.295±0.179 | 3.026±0.810 | | 3.381±0.125 | |  | 3.660±0.744 | | 3.716±0.345 | | 3.472±0.251 |
| *C. camphora* | 1.923±0.134 | | | 1.661±0.139 | | 2.050±0.179 |  | 2.985±1.065 | 3.315±0.381 | | 3.262±0.017 | |  | 3.660±0.303 | | 3.360±0.533 | | 3.206±0.242 |
| *A. confusa* | 2.194±0.181 | | | 1.879 ±0.085 | | 2.131±0.182 |  | 3.134±0.588 | 3.361±0.124 | | 3.486±0.327 | |  | 4.076±0.138 | | 4.040±0.189 | | 3.771±0.079 |
| *P. massoniana* | 1.744±0.447 | | | 1.727±0.174 | | 1.832±0.306 |  | 2.685±0.538 | 2.671±0.104 | | 3.465±0.231 | |  | 2.428±0.088 | | 2.532±0.340 | | 2.766±0.146 |
| *S. superba* | 1.810 ±0.535 | | | 1.659±0.339 | | 2.107 ±0.318 |  | 2.954±1.077 | 2.948±0.794 | | 3.082±0.129 | |  | 3.554±0.460 | | 3.537±0.113 | | 3.221±0.461 |
| *C. chinensis* | 1.685±0.632 | | | 1.546±0.169 | | 1.562±0.137 |  | 3.894±0.568 | 3.434±0.299 | | 2.893±0.588 | |  | 3.847±0.153 | | 3.926±0.119 | | 3.638±0.163 |
