# Supplementary material for: Non-Additive Effects on Decomposition from Mixing Litter of the Invasive Mikania micrantha H.B.K. with Native Plants
Source: PLoS One. 2013 Jun 20;8(6):e66289. doi: 10.1371/journal.pone.0066289 (PMC3688783; doi:10.1371/journal.pone.0066289)
Supplement: Appendix S1 — Methods of calculating N and C release. (DOCX) [file pone.0066289.s007.docx]

**Appendix S1 Methods of calculating N and C release**

Observed N and C release from single-species litter was calculated as:

N release from litter (g N/litterbag) = N*_il_*× M*_lb_* –N*_la_* × M*_la_* (1)

C release from litter (g C/litterbag) = C*_il_*× M*_lb_* – C*_la_* × M*_la_* (2)

*Where*, N*_il_* : Initial N content in litter, N*_la_* : N content in litter after decomposition, C*_il_* : Initial C content in litter, C*_la_* : C content in litter after litter decomposition, M*_lb_*: Litter mass in the litter bags before decomposition, M*_la_* : Litter mass remaining after decomposition.

Nutrient release was calculated as initial N and C content in the litterbags minus N and C content in the remaining litterbags after decomposition. Observed N release (ON*r*) and observed C release (OC*r*) from mixed-species litter were calculated as:

ON*r* (g N/litterbag) = (N*_inl_* × M*_nlb_* + N*_iel_* × M*_elb_*) - (N*_omla_*× M*_omla_*) (3)

OC*r* (g C/litterbag) = (C*_inl_* × M*_nl b_*+ C*_iel_* × M*_elb_*) - (C*_omla_*× M*_omla_*) (4)

*Where*, N*_inl_*: Initial N content in native litter, N*_iel_*: initial N content in exotic litter, N*_omla_*: observed N content in mixed litter after decomposition, C*_inl_*: Initial C content in litter, C*_iel_* : Initial C content in exotic litter, C*_omla_*: observed C content in mixed litter after decomposition, M*_nlb_*: native litter mass in the litter bags before decomposition, M*_nlb_*: native litter mass in the litter bags before decomposition, M*_elb_*: exotic litter mass in the litter bags before decomposition, M*_omla_*: observed mixed litter mass remaining after decomposition.

Predicted N release (PN*r*) and predicted C release (PC*r*) was calculated as:

PN*r* (g N/litterbag) = (N*_inl_* × M*_nlb_* + N*_iel_* × M*_elb_*) - (N*_onla_*× M*_onla_* + N*_oela_*× M*_oela_*) (5)

PC*r* (g N/litterbag) **=** (C*_inl_* × M*_nlb_* + C*_iel_* × M*_elb_*) - (C*_onla_*× M*_onla_* + C*_oela_*× M*_oela_*) (6)

*Where*, N*_inl_* , N*_iel_*, C*_inl_* , C*_iel_*, M*_nlb_* and M*_elb_* are same as the above. N*_onla_*: observed N content in single native litter after decomposition, N*_oela_*: observed N content in exotic litter after decomposition, C*_onla_*: observed C content in single native litter after decomposition, C*_oela_*: observed C content in exotic litter after decomposition.

M*_onla_*: observed single native litter mass remaining after decomposition, M*_oela_*: observed exotic litter mass remaining after decomposition.

Mixed strength of litter N and C release was calculated as

= 1- (Observed/Predicted N and C release from mixed litter)

Mixed strength of N release =1-ON*r*/PN*r* (7)

Mixed strength of C release =1-OC*r*/PC*r* (8)

In the absence of a mixture interaction, the value should be (close to) zero. Because we used the difference between the nutrient content of litter before and after decay to express litter nutrient release in the calculations, positive and negative interactions would yield values that are respectively greater or smaller than zero. Stronger mixture interactive effects would lead to a greater departure from zero (either positive or negative).
